# Changes in Speckle Tracking Echocardiography Values of the Descending Thoracic Aorta with Rising Positive End-Expiratory Pressure Levels

**DOI:** 10.3390/medicina61101865

**Published:** 2025-10-16

**Authors:** María Belén Martínez-Lechuga, Javier Hidalgo-Martín, José Ángel Ramos Cuadra, Julia Manetsberger, Ana Blanco-Serrano, Veronica Todaro, Gabriel Heras-La-Calle, María Leyre Lavilla Lerma, Juan Carlos Fernández-Guerrero, Manuel Ruiz-Bailén

**Affiliations:** 1 University Jaén Hospital, 23007 Jaén, Spain; mariab.martinez.sspa@juntadeandalucia.es; 2 Cardiac Critical Care, Intensive Care Medicine Department, University Hospital of Jaén, 23007 Jaén, Spain; javier.hidalgo.martin.sspa@juntadeandalucia.es (J.H.-M.); ana.blanco.serrano.sspa@juntadeandalucia.es (A.B.-S.); veronica.todro.sspa@juntadeandalucia.es (V.T.); gabriel.heras.sspa@juntadeandalucia.es (G.H.-L.-C.); 3 Intensive Care Medicine, Hospital Torrecárdenas, 04009 Almería, Spain; josea.ramos.cuadra.sspa@juntadeandalucia.es; 4 Department of Health Sciences, University of Jaén, Campus Las Lagunillas, 23071 Jaén, Spain; jmanetsb@ujaen.es (J.M.); llavilla@ujaen.es (M.L.L.L.); 5 Interventional Cardiology Unit, University Jaén Hospital, 23007 Jaén, Spain; juanc.fernandez.guerrero.sspa@juntadeandalucia.es

**Keywords:** PEEP, speckle tracking, aorta, cardiogenic shock

## Abstract

*Background and Objectives:* The aim of this study is to evaluate the changes in speckle tracking velocity vector analysis (VVI) values within the descending thoracic aorta (DTA) in patients with cardiogenic shock (CS) who are on mechanical ventilation (MV), under varying levels of positive end-expiratory pressure (PEEP). *Materials and Methods:* Transthoracic echocardiography (TTE) was performed during incremental increases in positive end-expiratory pressure (PEEP) from 0 to 15 cmH_2_O over 15 to 30 min. The effects of increased PEEP on velocities, displacement, strain (S), and strain rate (SR) were evaluated. DTA speckle tracking values were analyzed to determine their association with patient mortality. A control group of healthy individuals was used to establish normal DTA variables. *Results:* Sixty-two mechanically ventilated patients were included in this study. The mean age was 62.48 ± 11.22 years. The highest values for various parameters were obtained with 5 cmH_2_O PEEP. The values obtained for DTA using speckle tracking at increasing PEEP levels (ZEEP, PEEP 5, PEEP 10, and PEEP 15 cm H_2_O) were as follows: DTA rotational velocity [55.18 ± 14.60, 107.39 ± 19.33, 60.05 ± 0.28, and 42.11 ± 0.34°/s], DTA radial velocity [0.80 ± 0.09, 2.21 ± 0.27, 0.99 ± 0.16, 0.56 ± 0.17 cm/s], DTA rotational displacement [5.68 ± 0.40, 15.71 ± 0.13, 5.98 ± 0.35, 6.64 ± 3.45°], circumferential strain for DTA [−8.55 ± 0.92, −11.86 ± 0.07, −9.88 ± 0.25, −8.76 ± 0.6%], and DTA circumferential SR [−0.87 ± 0.1, −1.91 ± 0.03, −1.21 ± 0.12, −0.97 ± 0.05/s]; all *p*-values < 0.05. Logistic binary regression found left ventricular strain and DTA rotational displacement on 5 cmH_2_O PEEP level were associated with death. *Conclusions:* Changes in PEEP levels affect the speckle tracking measurements of the DTA. Speckle tracking can be used to assess the thoracic aorta, and certain parameters, such as rotational displacement, may relate to the prognosis of cardiogenic shock.

## 1. Introduction

The incidence of cardiogenic shock (CS) in acute myocardial infarction (AMI) remains at 10% [1], with mechanical ventilation (MV) required in 40–80% of cases [1]. Patients often experience cardiogenic edema with severe hypoxemia, necessitating distinct levels of PEEP to enhance oxygenation and increase lung recruitment. Previous research by our team indicated that varying levels of PEEP could alter ventricular strain and potentially improve mitral regurgitation [2]. Positive end-expiratory pressure (PEEP) is a well-studied intervention commonly used in ICUs. Its use causes significant hemodynamic changes, first noted last century. PEEP can alter extravascular lung water in models of pulmonary and cardiogenic edema, and patients frequently need positive-pressure ventilation (PPV) [3,4].

Cardiogenic shock mortality is often associated with the left ventricular ejection fraction. Nevertheless, this relationship remains intricate. Despite recent anatomical findings, like those by Torrent Guasp [5,6], cardiovascular pathophysiology still depends on key concepts: contractility, preload, afterload, ventricular interdependence, and diastology. However, the integration of atrial contribution, synchrony, and particularly aortic function remains unclear. Aortic function and its underlying mechanisms remain poorly understood, with limited current research available [7,8]. Systemic inflammation and neurohumoral factors mediate this process. Interestingly, studies indicate that muscle cells in the aorta and the use of levosimendan can enhance aortic relaxation [9]. Echocardiography has improved the diagnostic evaluation of hypoxemia in intensive care units, facilitating earlier identification of heart failure and potentially lowering the incidence of acute respiratory distress syndrome. As a result, echocardiography has become an indispensable tool for managing critically ill patients in intensive care settings [10], particularly in cases of cardiogenic shock, which continues to be associated with high mortality rates [11,12,13,14,15]. Speckle tracking has been employed in all four cardiac chambers, yielding diagnostic data. Previous research, however, has not examined the use of this technique for the aorta. Recently, our research team implemented speckle tracking velocity vector analysis to evaluate the velocity, deformation, and displacement of the descending thoracic aorta (DTA) [7].

Speckle tracking echocardiography has been used in research with mechanically ventilated patients [2,5]. Although the cardiovascular effects of PEEP are well documented, the extent of alterations occurring within the aorta remains undetermined. This variability may be attributable to changes in transmural pressure, variations in ventricular filling, or inherent characteristics of the aorta itself. Furthermore, aortic measurements have potential utility as parameters for evaluating optimal PEEP based on cardiovascular responses. To date, no published studies have applied speckle tracking to the aorta at varying levels of PEEP.

This study examined the effects of increased PEEP levels on strain, strain rate, radial and circumferential velocities, and displacements using speckle-tracking vector velocity imaging (VVI) echocardiography in critically ill CS patients on MV. The study also assessed whether speckle tracking VVI values are independently associated with mortality in cardiogenic shock.

## 2. Materials and Methods

### 2.1. Study Design

The present study was a prospective observational clinical study. The cohort was generated from all CS patients admitted in ICU who required MV. Rising levels of PEEP were applied to these patients. Changes in levels of PEEP are usually performed on critically ill patients on MV. CS was defined by the criteria used in the shock trials and specifically in the presence of systolic blood pressure below 90 mmHg that did not respond to volume administration, requiring the use of noradrenaline or adrenaline for at least 1 h [10,11,12,13]. This work was supported by PAIDI CTS 606, Andalusian Health Service Project no, “PI-0585-2012 Ecocardiografía en Medicina Crítica. Detección de la disfunción Miocárdica del Paciente Crítico e interacción entre la ecocardiografía y la ventilación mecánica Servicio Andaluz de Salud. Junta de Andalucía. España”; approved by the local ethics committee and funded by the Department of Health of the regional government of Andalusia, Spain. The data were stored in a dissociated database to ensure the blinding of the study. The present study was limited to Jaén Hospital. The inclusion period was from January 2014 to January 2024, excluding the years 2020 to 2022 due to the COVID-19 pandemic.

Clinical and demographic details were considered. All echocardiographic parameters recommended by the American Society of Echocardiography were duly considered (https://www.asecho.org/).

### 2.2. Study Protocol

This study is an echocardiographic investigation intended to evaluate aortic response to different levels of PEEP, rather than serving as an epidemiological analysis. As the focus is on echocardiography, information on clinical, hemodynamic, or laboratory variables is limited; interleukin-6 was the only marker consistently measured in all mechanically ventilated patients (Kit Elecsys IL-6, (Bio-Techne, Minneapolis, MN, USA) detection limit of 1.5 pg/mL cobas^®^). Troponin and brain natriuretic peptide were not measured due to changes in testing methods during the study period.

### 2.3. Inclusion Criteria

The following criteria were applied for the inclusion of patients in the study: (a) patients with ST elevation myocardial infarction (STEMI) who develop CS and have required norepinephrine and MV; (b) having been revascularized by percutaneous cardiovascular intervention; (c) PEEP ≥ 5 cmH_2_O; (d) FiO_2_ ≤ 0.5; and (e) ventilatory plateau pressure ≤ 25 cmH_2_O.

### 2.4. Exclusion Criteria

Patients were excluded from the study for any of the following reasons: (1) having been managed without echocardiography; (2) poor sound quality; (3) requiring a certain level of PEEP specific to the severity of their condition; (4) refusing to participate in the study; (5) having CS of etiology other than acute coronary syndrome; (6) patients who were transferred to a center with a cardiovascular surgery service (not available in our center); (7) patients who only required the use of noradrenaline during percutaneous coronary intervention, arriving at the coronary unit without vasoactive amines; (8) mechanical complications (6 ventricular septal defects, 8 papillary muscle ruptures, 3 functional mitral insufficiencies requiring surgical repair and 12 cardiac ruptures); (9) aortic valvulopathy ≥ grade 3 and (10) aortic atheroma > 2 Kazt’ grade classification [14].

### 2.5. Changes in PEEP Levels

Patients requiring varying levels of PEEP as a routine part of the management of their condition were included in the study. All patients were started on ZEEP, and the PEEP was increased by 5 cmH_2_O, until a maximum PEEP of 15 cmH_2_O (ZEEP, PEEP 5, PEEP 10, and PEEP 15 cmH_2_O). This procedure was performed after hemodynamic stability had been achieved and after removing all vasoactive drugs (epinephrine, norepinephrine, dobutamine, and levosimendan), although the entry criterion was to have been admitted with CS. No other breathing pattern was changed. Echocardiography was performed after 15 to 30 min of introducing changes in PEEP levels. Patients were without respiratory drive or were sedated to abolish respiratory drive. Modification of PEEP levels was suspended if the patient showed signs of clinical deterioration, such as (a) decrease in saturation oxygenation, (b) increase in peak or plateau pressures, (c) impairment of protective MV, or (d) hemodynamic changes amounting to 10% (changes in blood pressure or heart rate).

### 2.6. Control Group

A control group of healthy subjects without CS and with spontaneous breathing was included to establish reference values for our coronary unit via speckle tracking of the DTA. They were compared to CS patients on MV with a PEEP of 5 cmH_2_O, as most patients on MV had at least that level of PEEP.

### 2.7. Image Acquisition and Processing

Patients underwent an advanced TTE, which was conducted in supine position and was recorded in digital Dicom format. For off-line analysis purposes, we used 2D and 3D and Speckle Tracking Hybrid VVI [15]. Echocardiographic studies were performed with the following echocardiographic equipment available during long-term study period: Sequoia c512, Acuson, SC 2000, Siemens Medical Systems, Mountain View, CA, USA, and Epiq CX50. TTE was performed in the control group and in all patients. Transoesophageal echocardiography (TEE) was only performed when the diagnosis of shock was not clear and mainly to evaluate the mitral valve or when there was suspicion of mechanical complications. were realized. The echocardiographic variables studied have been determined both online and offline, using echocardiographic programs Syngo software 2013, Siemens^®^ U.S. and Tomtec Arena 2020 (https://www.tomtec.de/excellence-in-digital-healthcare/, accessed on 10 October 2024).

The apical four-chamber view was utilized to obtain data on left ventricular function and the DTA analysis. The frame rate varied from 70 to 120 frames per second, incorporating multiple focal points. All images were meticulously adjusted for gain, compression, and dynamic range.

The standard echocardiographic parameters, as recommended by the American Society of Echocardiography, were evaluated, including the quantification of left ventricular systolic ejection fraction (LVEF) using both 2D and 3D methods. Additionally, parameters derived from speckle tracking echocardiography, such as strain (S), strain rate (SR), longitudinal and radial displacement, and velocities in the left ventricle, were assessed. Furthermore, we analyzed radial and rotational velocity, radial and circumferential strain, strain rate, as well as rotational and radial displacement in the descending thoracic aorta (DTA) using Speckle Tracking Vector Velocity Imaging (VVI) analysis. The region of interest was meticulously delineated manually.

### 2.8. Statistical Analysis

The Kolmogorov–Smirnov test was used to assess the normality of the variables. After confirming the normality of the variables, quantitative variables were analyzed using ANOVA and Student’s *t*-test. The Levene statistic was used to evaluate variance homogeneity. When equal variances were assumed, the Bonferroni test and Tukey post hoc test were applied to control the probability of Type I error. The Anova test was used to evaluate the changes in the different clinical and echocardiographic variables with different PEEP levels. Student’s *t*-test was employed to assess the differences between the control group and the group treated with 5 cmH_2_O of PEEP, as this level of PEEP is the most used. The correlation between left ventricle ejection fraction, left and right ventricular strain, and DTA variables were studied using Pearson’s correlation coefficient. The degree of agreement between the strain values obtained in the parasternal long axis and the apical four-chamber planes was evaluated using Bablok regression and a Bland–Altman plot. Results were presented using means and standard deviations. A *p* value < 0.05 was considered statistically significant.

A multivariate logistic regression analysis was conducted, with “deceased” designated as the dependent variable and only echocardiographic data serving as independent variables. Statistical analyses were performed using IBM SPSS version 30 (Chicago, IL, USA).

## 3. Results

### 3.1. Cardiogenic Shock Patients

A total of 1893 STEMI patients were included during the study period, 158 developed CS (8.4%), 94 (59.49%) required MV. We excluded 15 patients admitted for apparent septic shock and acute respiratory failure initially attributed to possible infections, who were later shown to have suffered an AMI. Six patients with a mean age of 52.38 ± 18.32 years were transferred to a different hospital for extracorporeal membrane oxygenator (ECMO) implantation, after implantation of balloon counterpulsation or impella catheter and maintained in SCAI D shock; they were also excluded. Eleven patients initially included in the study, recovered from CS, presented weaning failure with grade 2–3 functional mitral regurgitation and normal LVEF. All of them underwent stress TEE and grade 4 mitral regurgitation was observed. They were transferred to mitral valve surgery and excluded. One of those patients who was excluded because she was initially diagnosed with sepsis, had a thrombus in the thoracic aorta seen on TEE. She had thoracic aortic velocities, displacements and strain of almost 0, and there was no change with increases in PEEP level.

**Figure 1:**
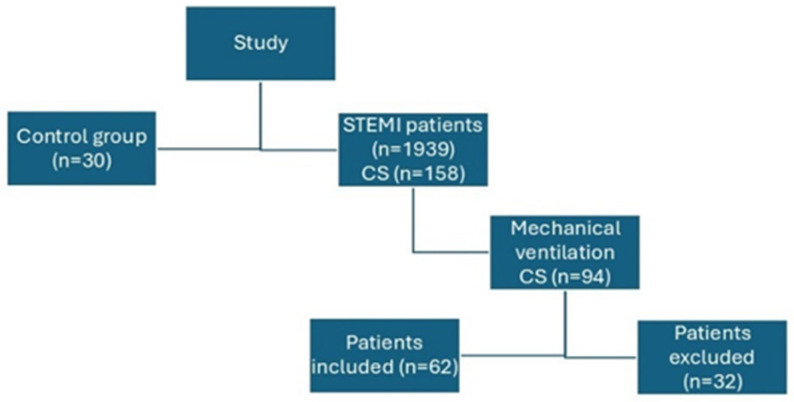
Study design. This study involved two cohorts: a control group of thirty healthy individuals for reference parameters, and a patient group of 1939 STEMI cases enrolled during the study period. Of these patients, 158 developed cardiogenic shock and ninety-four required mechanical ventilation. Following the exclusion of thirty-two patients from the analysis, the final cohort consisted of sixty-two individuals with cardiogenic shock on mechanical ventilation; among this group, twenty-eight did not survive.

A total of sixty-two patients with CS were enrolled in the study (Figure 1), of whom 29 (46.78%) were female. Risk factors and complications are detailed in Table 1. The mean APACHE II score was 22.91 ± 13.45. The average LVEF measured 27.12 ± 0.015% and the lateral right ventricular strain was −15.64 ± 0.11. The mean age was 62.48 ± 11.22 years, and the mean ICU length of stay was 15.32 ± 12.22 days. In total, 28 patients (45.61%) died.

The mean Interleukin-6 (IL-6) value among the 62 patients requiring mechanical ventilation was 137.12 ± 5.29 pg/mL, which, in bivariate analysis, was associated with increased mortality (84.89 ± 7.33 pg/mL in survivors versus 165.06 ± 11.17 pg/mL in deceased patients; Student’s *t*-test, *p* < 0.001). IL-6 levels correlated with DTA rotational displacement (R^2^ = 0.39, *p* = 0.001). Hemodynamic and ventilatory parameters are presented in Table 2.

### 3.2. Changes in PEEP Levels

With increasing PEEP level from 0 to 15 cmH_2_O increased SpO_2_, peak and plateau pressures, but no clear changes in hemodynamic variables were observed. With this increase in PEEP levels there was a significant increase in left ventricular strain and strain rate, although LVEF also seemed to increase, although not significantly. Right ventricular function did not differ significantly. With increasing PEEP, the delay times between the different ventricular segments in the strain and strain rate decreased, and there may be greater synchrony in the left ventricle with increasing PEEP (Table 3).

### 3.3. Descending Thoracic Artery Aorta

DTA artery mean diameter was 27.82 ± 3.45 mm with no substantial changes. However, there were very significant changes in its fractional shortening area, rotational velocity, radial velocity, strain, strain ratio, and rotational displacement. The radial displacement increases greatly with 5 cmH_2_O but drops to ZEEP levels with further increases in PEEP, with no statistical significance. In all these parameters generated by speckle tracking using VVI analysis a clear pattern could be observed. When treating patients with 0 cmH_2_O of PEEP (ZEEP) the minimum value is obtained in all analyzed values, and after applying 5 cmH_2_O the maximum increase in all values is produced, especially in their rotational velocity (which increases by double).

**Figure 2:**
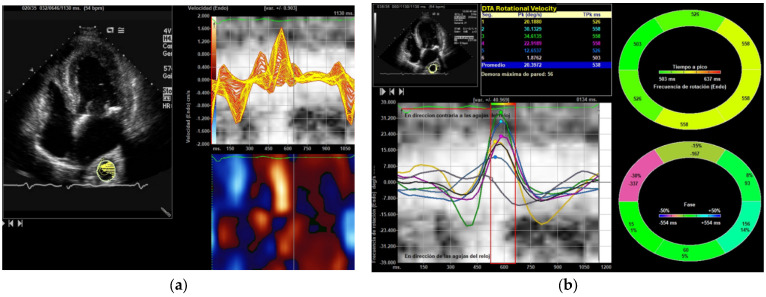
(**a**,**b**) DTA rotational velocity is measured using the apical four-chamber view. Parasternal and apical views imaging projections are suitable for Speckle Tracking VVI analysis. The various values throughout the cardiac cycle can be assessed; in this study, we evaluated the average of the distinct systolic peaks.

**Figure 3:**
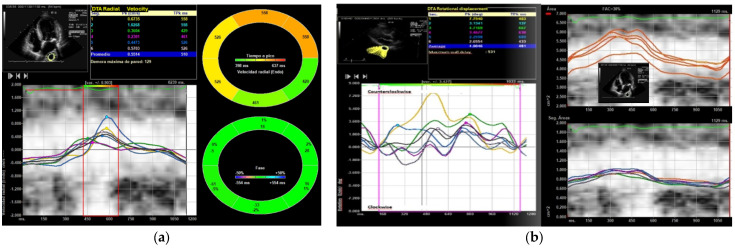
(**a**) This figure shows the radial velocity of a DTA from a patient with cardiogenic shock, where the average of the different systolic peaks in cm/s is obtained using an apical four-chamber view. (**b**) This figure displays the DTA rotational displacement measured in the parasternal view. The average of the various systolic peaks is quantified. This figure also illustrates the fractional shortening area of the aorta (%).

After raising the PEEP level to 10 cmH_2_O almost all its parameters, except for the two types of displacement, continued to be higher than the initial value, and after increasing to 15 cmH_2_O, the values returned to those obtained without PEEP or slightly higher. Interestingly, after applying ≥10 cmH_2_O we observed an increased loss of echocardiographic DTA due to the worsening of the acoustic window and the impossibility of speckle tracking use. The DTA rotational displacement correlates with left ventricular longitudinal strain, right ventricular longitudinal strain, but not with LVEF assessed by 2 and 3D echocardiography (Pearson’s r 0.32, and 0.41, respectively, *p* < 0.001). DTA rotational velocity correlate only with left ventricular strain (Pearson’s r 0.26, *p* < 0.001). Logistic binary regression identified left ventricular strain [OR 0.75 (95% CI 0.45–0.91), *p* < 0.05], rotational velocity [OR 0.38 (95% CI 0.12–0.88), *p* < 0.05], and rotational displacement [OR 0.55 (95% CI 0.38–0.85), *p* < 0.05] as echocardiographic variables associated with death in the DTA group with 5 cmH_2_O of PEEP (Figure 2 and Figure 3).

Patients in the control group had higher diastolic pressure, lower respiratory rate, higher ejection fraction, higher systolic velocities on tissue Doppler imaging of the mitral annulus, greater left ventricular deformity, better right ventricular function, but nevertheless with the use of 5 cmH_2_O of PEEP the speckle tracking values of the thoracic aorta increased (Table 4).

## 4. Discussion

This echocardiographic study attempts to evaluate the changes produced in the parameters derived from speckle tracking by VVI analysis in the DTA in patients in CS requiring MV. Although the work also provides some epidemiological data on CS, we do not intend to perform an epidemiological study, as we lack crucial clinical and angiographic variables to be able to make any assertion on CS and the study design is not appropriate for this purpose. Incidence appears to be lower than that reported in the Spanish ARIAM registry [16,17] and other registries [1]. Spanish registry in 2011 [18] we already perform echocardiography in almost 100% of patients with CS, and most patients are followed up nearly daily.

The mean age of patients in this study is lower than the ages reported in the ARIAM registry [14], the RESCUE registry (66 years) [19], the Shock registry [11], CardShock (68.7 years) [20], and several other registries [21,22,23,24,25,26,27]. The rate of MV in this study was lower than that in the RESCUE registry, but within the previously described ranges for CS (40 to 80%) [28].

The average length of stay observed in this study is comparable to findings from other registries, particularly the RESCUE study (12 days) [19]. Observed mortality rates were lower than those reported in the ARIAM registry [14] and the Shock registry (61%) [11], but higher than those found in the Korean RESCU register (33.6%) [19] and CardShock (36.5%) [20].

In summary, the population included in this echocardiographic study is like that of other registries. Interestingly, we have performed stress echocardiography to detect weaning failure and severe valvular heart disease; an option that we consider very valid in critically ill patients in whom the degree of mitral regurgitation is in doubt or in the presence of weaning failure [29,30].

PEEP—and positive-pressure ventilation in general—has been shown to improve ventricular function [2]. Furthermore, and although it might be a false response to transmural pressure changes, benefits in left atrial remodeling have been shown in patients with sleep apnea hypopnea syndrome [31,32]. One finding that confirmed previous results from by our group showed that PEEP could improve left ventricular synchrony, which would translate into an improvement of significant left ventricular function [19]. Speckle tracking could predict the cardiological evolution of sleep apnea-hypopnea syndrome [33] and could even decrease latent systolic dysfunction in these patients [34].

The most interesting result of this study is that we have been able to quantify the values of the DTA in CS patients and that these values have also varied with the levels of PEEP introduced. This represents a new avenue for research on the interaction between aorta and MV; but it also implies that its values are associated with mortality in CS. Interestingly, this study may suggest that evaluating DTA values could indicate the optimal PEEP level and serve as a prognostic marker in shock scenarios. In CS and heart failure more broadly, numerous variables affect the response; however, aortic pathology is typically not considered as a predictor variable. The aorta can be divided into three parts: ascending aorta, aortic arch and descending aorta. Primarily, its functions involve opening and closing the aortic valve and assisting with ventricular ejection [35]. An important challenge involves evaluating this activity, with diameter measurements frequently serving as indicators of remodeling [36]. Cardiac resonance is essential for identifying physiological changes [37]. Echocardiography is limited to detecting diameters, atheromatous plaques, remodeling, or complications such as acute aortic syndrome. Speckle tracking is a fundamental tool in cardiology; however, there is no software that uses this technology to evaluate the aorta [38]. VVI speckle tracking was applied in the thoracic aorta to establish baseline measurements and monitor changes in velocities, strain, strain rate, and DTA displacement at different levels of PEEP. However, in the multivariable analysis, only rotational displacement was identified as a variable associated with mortality. Also, rotational displacement correlated with LV and RV longitudinal strain and LV strain rate.

The explanation is still unknown, but we believe that what is being measured is the behavior of the aorta, and that rotational displacement is probably a more precise parameter than its velocities (especially radial velocity) or its strain. Undoubtedly, we consider it a strong and potentially accurate parameter.

The observed correlation may be attributable to the anatomical linkage between the heart and the aorta, potentially facilitating the transmission of cardiac motion and flow into the aortic vessel. Additionally, these findings may also reflect both the physiological state and aorta remodeling.

The value associated with mortality is DTA rotational displacement at 5 cmH_2_O PEEP, suggesting this may represent the optimal level. Alterations in aortic geometry resulting from transmural pressure and aortic muscle activity could account for these findings. A PEEP less than 10 cmH_2_O may be advantageous for aortic function, whereas 15 cmH_2_O might be harmful, thereby assisting in identifying the most appropriate PEEP setting.

Therefore, we believe that aortic assessment could have prognostic value in certain pathologies or shock situations. Previous studies have reported that patients with aortic atherosclerosis or a rigid aorta may exhibit symptoms of heart failure, independent of their underlying cardiovascular condition [38,39]. The aorta plays a key role in cardiovascular function and warrants further study. Speckle tracking may improve our understanding of its physiology. Heart failure after aortic repair could demonstrate this reciprocal relationship [40]. This is exploratory work, but it implies that specific software based on speckle tracking could be developed to assess aorta function.

The results may also be conditioned by the degree of inflammation [41,42,43]; in fact, the present study shows that interleukin 6 is related to displacement and rotational aorta. Other authors have suggested anti-inflammatory drugs to limit the severity of aortic pathology [43].

Pathological aortic remodeling could affect not only the aortic valve but also the entire cardiovascular pathophysiology. Another variable (although not used in this study) which could provide information on the hemodynamic situation is the time velocity integral in the abdominal aorta. This value could be correlated with the circulation at that level and the hemodynamic situation. It is an easy measurement, evaluable in the subcostal plane.

### Limitations

One limitation of this study is the lack of specific software designed for aortic assessment using speckle tracking. While VVI speckle tracking was employed to monitor changes in the thoracic aorta, current echocardiographic tools are limited and do not fully capture the complexity of aortic motion or remodeling [7]. The different systolic peaks are determined; the software is intended for speckle tracking of the ventricular mass but is used on the aortic wall, which could lead to issues. Nonetheless, credible patterns have emerged that may demonstrate improved safety and efficacy when assessed with software specifically developed for the aorta. Therefore, while the study presents an innovative concept, its findings should not be widely extrapolated. It is appropriate only to propose the hypothesis that Speckle Tracking could be applied to evaluate the aorta, and that the resulting values may correlate with outcomes in cardiogenic shock. However, our results are not generalizable. Further research and technological advances may include the development of specialized aortic analysis software, enabling more precise assessment of the vessel’s dynamic properties in both healthy individuals and CS patients.

Here, we used a small sample of patients with CS, which may limit patient selection and introduce potential bias. Furthermore, the changes detected with PEEP demonstrated patterns that indicate possible alterations in aortic physiology and may warrant further investigation regarding its relevance for determining optimal PEEP levels at the cardiovascular level. Additionally, as this study is exploratory, its findings should be validated in larger patient groups and under varying clinical conditions.

Although there are limitations, this study indicates that incorporating speckle tracking in the aorta may offer valuable insights. Establishing reference values could facilitate a better understanding of aortic pathologies and cardiovascular interactions, potentially contributing to the interpretation of heart failure semiology.

## 5. Conclusions

Increasing PEEP affects DTA speckle tracking values. Speckle tracking can assess the thoracic aorta, and parameters like rotational displacement may relate to cardiogenic shock prognosis. Further research into aorta-specific speckle tracking technology is recommended.

Speckle tracking VVI has not previously been used to assess the aorta; this study applies it to the DTA, comparing values in healthy individuals and those with cardiogenic shock. The results show that these values vary with mechanical ventilation settings and may be linked to mortality in cardiogenic shock.

## Figures and Tables

**Table 1:** Comorbidities and complications among patients: Anterior infarction was the most common cause of CS, and high blood pressure was the leading risk factor. Patients showed similar risk factors and comorbidities to those reported in other registries.

Patients	CS People (n [%])
**Acute myocardial infarction location**	
Anterior	40 (64.51)
Inferior	22 (35.48)
**Comorbidity**	
Pulmonary disease	12 (19.35)
Hypertension	38 (61.29)
Diabetes	28 (45.16)
Hypercholesterolemia	24 (38.71)
Tobacco use	18 (29.03)
Previous Acute Coronary Syndrome	31 (50%)
**Complications**	
Infectious process	18 (29.03)
Ventricular fibrillation	19 (30.64)

**Table 2:** Hemodynamics and ventilatory variations according to the PEEP level used.

Variables	ZEEP	PEEP 5	PEEP 10	PEEP 15	*p*
**Systolic blood pressure (mm Hg)**	115.26 ± 14.08	112.88 ± 11.21	100.45 ± 9.161	94.14 ± 5.31	0.032
**Diastolic blood pressure (mm Hg)**	56.45 ± 6.88	51.32 ± 5.54	62.37 ± 3.38	41.32 ± 4.31	NS
**Heart rate (bpm)**	96.52 ± 3.27	101.21 ± 6.01	104.32 ± 9.41	105.38 ± 3.21	NS
**Respiratory rate**	21.18 ± 10.15	21.18 ± 10.15	21.18 ± 10.15	21.18 ± 10.15	NS
**SpO_2_ (%)**	91.90 ± 0.75	95.44 ± 0.22	98.77 ± 0.28	97.88 ± 0.14	0.001
**Dynamic resistance (cm H_2_O/L/s)**	24.47 ± 1.37	19.99 ± 0.88	15.71 ± 0.88	16.32 ± 0.23	0.001
**Plateau pressure (cm H_2_O)**	24.32 ± 3.17	21.18 ± 09.8	28.32 ± 0.71	34.44± 0.71	0.028
**Peak pressure (cm H_2_O)**	36.22 ± 0.13	37.28 ± 0.12	41.33 ± 0.89	45.38 ± 0.19	0.0011
**Static compliance (mL/cm H_2_O)**	33.11 ± 0.115	44.15 ± 09.32	42.24 ± 1.59	32.27 ± 1.32	0.04
**AutoPEEP (cm H_2_O)**	5.31 ± 0.22	4.98 ± 03.73	7.25 ± 03.10	9.88 ± 0.719	0.01

Anova’s test was used. ZEEP. = 0 cmH_2_O. PEEP (cmH_2_O). NS indicates not significant.

**Table 3:** Systolic Function Parameters.

Variables	ZEEP	PEEP 5	PEEP 10	PEEP 15	*p*
**LVEF 3D (%)**	36.45 ± 0.17	46.54 ± 0.34	47.21 ± 0.09	46.02 ± 0.032	NS
**LVEF 2D (%)**	27.43 ± 0.015	34.21 ± 0.21	41.69 ± 0.083	45.15 ± 0.12	NS
**Septal wave S velocity (m/s)**	0.059 ± 0.0054	0.062 ± 0.0058	0.068 ± 0.0024	0.065 ± 0.0087	NS
**Basal-lateral wave S velocity (m/s)**	0.068 ± 0.0044	0.076 ± 0.0021	0.078 ± 0.0032	0.045 ± 0.0097	NS
**VTI in LVOT (cm)**	9.21 ± 0.02	11.77 ± 0.07	12.91 ± 0.04	8.33 ± 0.09	0.021
**LVGLS (%)**	−11.27 ± 0.188	−14.37 ± 0.375	−16.31 ± 0.57	−14.88 ± 0.53	0.0032
**LVGLSR (1/s)**	−0.96 ± 0.04	−1.04 ± 0.24	−1.36 ± 0.28	−1.28 ± 0.44	0.0001
**LV Longitudinal Strain delay (ms)**	285.57 ± 14.034	156.54 ± 32.54	134.89 ± 11.01	245.88 ± 17.88	0.0001
**LVGLSR delay (ms)**	155.32 ± 13.27	132.45 ± 11.32	110.87 ± 14.28	134.72 ± 17.09	0.0001
**Lateral right S’ wave (m/s)**	0.079 ± 0.0054	0.087 ± 0.0034	0.088 ± 0.0024	0.085 ± 0.0087	NS
**TAPSE (mm)**	16.98 ± 0.23	17.23 ± 0.12	18.44 ± 0.19	17.77 ± 0.12	NS
**RVGLS**	−18.22 ± 0.24	−21.12 ± 0.28	−17.11 ± 0.18	−9.34 ± 0.74	0.014

Anova’s Test were used. LVEF: left ventricular ejection fraction. ZEEP. = cmH_2_O. PEEP (cmH_2_O). NS indicates not significant. LVOT: Left ventricular outflow tract. LVGLS. Left ventricular global longitudinal strain (%). Left ventricular global strain rate (1/s) RVGLS. Right ventricular global longitudinal Strain (%). VTI: Velocity integral time (cm).

**Table 4:** Differences between the control group and patients undergoing mechanical ventilation with 5 cmH_2_O. Only this PEEP level was evaluated, as it was the lowest PEEP level that patients had and the most used. The aim was to see the differences between the control group and a PEEP level that, although not optimal, was the most used in intensive care medicine. N.S. is not statistically significant.

Variables	Control Group (n = 30)	PEEP 5 (n = 62)	*p* Value
**Systolic arterial pressure (mm Hg)**	122.32 ± 0.89	112.88 ± 20.21	N.S.
**Diastolic arterial pressure (mm Hg)**	72.85 ± 09.17	51.32 ± 14.52	0.03
**Heart rate (bpm)**	72.32 ± 11.37	101.21 ± 18.34	<0.001
**Respiratory rate**	15.42 ± 7.22	21.18 ± 10.15	<0.001
**SpO_2_ (%)**	96.32 ± 0.97	95.44 ± 5.22	N.S.
**LVEF 3D (%)**	66.32 ± 0.09	46.12 ± 0.34	<0.001
**LVEF 2D (%)**	5.289 ± 0.12	34.23 ± 0.21	<0.001
**Septal S wave velocity (m/s)**	0.10 ± 0.032	0.062 ± 0.058	<0.001
**Basolateral S wave velocity (m/s)**	0.12 ± 0.037	0.076 ± 0.021	<0.001
**LVGLS (%)**	−21.87 ± 2.15	−14.37 ± 3.75	<0.001
**LVGLSR (1/s)**	−1.64 ± 0.28	−1.04 ± 0.24	<0.001
**LV Strain delay (ms)**	122.32 ± 12.18	156.54 ± 32.54	N.S.
**LV Strain Rate delay (ms)**	144.57 ± 21.18	132.45 ± 51.21	N.S.
**Right lateral wave S’ velocity (m/s)**	0.12 ± 0.037	0.087 ± 0.034	<0.001
**FSA (%)**	0.56 ± 0.28	0.52 ± 0.18	N.S.
**TAPSE (mm)**	24.18 ± 2.01	17.23 ± 0.12	<0.001
**DTA Rotational velocity (°/s)**	54.44 ± 11.22	107.39 ± 0.19	<0.001
**DTA Radial Velocity (cm/s)**	1.18 ± 0.34	2.21 ± 0.27	<0.001
**DTA Circumferential Strain (%)**	−10.23 ± 0.45	−11.86 ± 0.07	N.S.
**DTA Strain Rate Circumferential (1/s)**	−1.67 ± 0.56	−1.91 ± 0.25	N.S.
**DTA Rotational displacement (°)**	7.38 ± 1.87	15.71 ± 0.13	<0.001
**DTA Radial displacement (mm)**	0.99 ± 0.32	1.58 ± 0.15	<0.022
**RVGLS (%)**	−26.25 ± 1.78	−21.12 ± 0.285	<0.001

T Students’ test was used. FSA Fractional shortening area DTA. LVGLS. Left ventricular global longitudinal strain.

## Data Availability

Supporting data is available. There are articles related to this manuscript in PubMed and Google Scholar. The database belongs to the Andalusian health system.

## Abbreviations

The following abbreviations are used in this manuscript:

3D	3 Dimensional
AMI	Acute myocardial infarction.
ASE	American Society of Echocardiography
AUC	Area under ROC curve
CS	Cardiogenic Shock
DICOM	Digital Imaging and Communication in Medicine
DTA	Descending Thoracic Aortic
DVD	Digital Versatile Disc
ECMO	extracorporeal membrane oxygenation
ESC	European Society of Cardiology
HF	Heart Failure
ICU	Intensive care medicine
IVA	Isovolumetric acceleration contraction
LA	Left atrial.
LDLV	Longitudinal displacement of left ventricle by Speckle Tracking
LDRV	Longitudinal displacement of right ventricle by Speckle Tracking.
LV	Left ventricle
LVEF	Left ventricle ejection fraction
LVGLS	Left ventricular global longitudinal strain
LVLV	Longitudinal velocity of left ventricular by Speckle Tracking
LVOT	VTI Left ventricle outflow tract—velocity time integral
LVRV	Longitudinal velocity of right ventricular by Speckle Tracking
MR	Mitral regurgitation
MRI	Magnetic resonance imaging
MV	Mechanical Ventilation
OR	Odds Ratio
PAIDI CTS 606	Estudios de las Cardiopatías Agudas. (Plan Andaluz de Investigación, Desarrollo e Innovación). Andalusian planning for investigation, development and innovation.
PCWP	Pulmonary capillary wedge pressure
PEEP	Positive end expiratory pressure
PPV	Possitive-pressure ventilation.
RDLV	Radial displacement of left ventricle by Speckle Tracking
RDRV	Radial displacement of right ventricle by Speckle Tracking.
RLAC	Right left atrial communication. Atrial septal defect and right- left shunt was confirmed by Serum agitated test or Sonovue^®^ administration.
RA	Right atrial
ROC	Receiver Operating Characteristic
RV	Right ventricle
RVEF	Right ventricular ejection fraction
RVGLS	Right ventricular global longitudinal strain
RVGSR	Right ventricular Global longitudinal strain rate (s^−1^)
RVLV	Radial velocity of left ventricular by Speckle Tracking
RVOT VTI	Right Ventricular outflow tract velocity-time integral
RVOTSF	Right ventricular outflow tract shortening fraction
RVV	Right ventricular velocity by Speckle tracking
S	wave (cm/s)
S’	wave velocity in the tricuspid annulus by tissue imaging
SBP	Systolic blood pressure
SpO_2_	Oxygen peripheric Saturation
TAPSE	Tricuspid annular plane systolic excursion
TEE	Transesophageal echocardiography
TR	Tricuspid regurgitation
TTE	Transthoracic echocardiography
